# Should Love be Explicitly Stated as a Core Enabling Concept in the Medical Curriculum?

**DOI:** 10.1177/23821205251408337

**Published:** 2026-03-27

**Authors:** Colin P Doherty, Claire L Donohoe

**Affiliations:** 1 School of Medicine, 8809 Trinity College Dublin, Dublin 8, Dublin, Ireland

**Keywords:** medical education, humanism in medicine, compassion, professional identity formation

## Abstract

The concept of love, defined as a multidimensional ethical commitment to human dignity encompassing empathy, compassion, and respect, should be explicitly integrated into the medical curriculum. This commitment is considered the “secret of quality” (Donabedian) and a necessary counterbalance to the reductive, metrics-driven model of modern healthcare. Acknowledging the risk of love becoming a narcissistic illusion (Lacanian critique), its implementation requires strict professional boundaries, “Self-as-Instrument” training via reflective practice and humanistic assessment over measurable metrics.

The concept of love, though rarely explicitly mentioned in modern medical education, holds profound significance for the alleviation of human suffering and should be central to the mission of any medical school. Avedis Donabedian, a renowned figure in healthcare quality and safety, made an important observation towards the end of his life: while systems design and process monitoring are essential, they fall short without the ethical commitment of individuals. “Ultimately, the secret of quality is love,” Donabedian concluded.^1^ This realisation speaks to the necessity of love not only as a guiding principle for the healthcare profession but also as a fundamental component of medical education. Love, in this context, is not a mere emotion but a multidimensional concept that encompasses empathy, compassion, and respect. It transcends the transactional nature of healthcare to create a human connection that is vital for healing.

## Defining Love in Medicine

Love, as an enabling concept, creates an environment where suffering can be alleviated because it centres on the human dignity of both the patient and the physician. Compassion, defined as “the recognition, understanding and emotional resonance with another's concerns… or suffering coupled with relational action to ameliorate these states”^2^ is an important value in healthcare. While empathy and compassion are often cited as essential qualities,^3^ they function as necessary but insufficient conditions for the full scope of the commitment we term *love* in this paper. Love is a more encompassing notion. It includes empathy (the capacity to understand another's experience) and compassion (the emotional response to suffering coupled with a desire to help), but also embraces vulnerability, self-respect, and mutual regard, transforming these individual traits into a holistic and relational ethos. Love fosters a holistic relationship that goes beyond the merely fiduciary one that exists and has always existed between the patient and Doctor; it touches on the broader dimensions of human experience, not simply the clinical interactions. While love is inherently holistic, its significance extends beyond this singular dimension. Therefore, in our framework, love is the overarching virtue that mobilises and directs the traits of empathy and compassion. It is the commitment to the human dignity of the other that provides the ethical foundation and sustained motivation for these specific emotional and relational skills.

One might argue that introducing love as a core concept in medical education challenges the rationalist and reductive model that currently dominates healthcare. In many ways, this is precisely why it should be included. The focus of modern medicine on empirical evidence, data, and outcomes has undoubtedly led to significant advances in diagnostics, therapeutics, and digital health. However, this same focus can often reduce patients to numbers and metrics, overshadowing the humanity at the heart of medicine.^4^ Love, as a counterbalance, reintroduces the personal dimension that is often missing. It reminds healthcare providers that they are treating individuals, not just diseases, and that the success of medical interventions is not measured solely in clinical outcomes but also in the alleviation of suffering and the restoration of dignity. When applied as a core value in clinical practice, this approach moves beyond the reductionism of evidence-based medicine. It embraces individual complexity, thereby harmonising the objectives of both cure and care.

Moreover, love in healthcare extends beyond the individual patient-provider relationship to encompass families and communities. A healthcare provider who practices with love recognises the interconnectedness of human beings and the social determinants that influence health outcomes. They understand that the alleviation of suffering cannot be achieved solely through clinical interventions but also requires addressing the broader context in which patients live. Love fosters a sense of social responsibility and motivates healthcare providers to advocate for policies and systems that promote health equity and justice. By including love in medical education, we can cultivate a generation of healthcare professionals who are not only skilled clinicians but also compassionate advocates for their patients and communities.

## Challenges to Inclusion

Critics might argue that love is a subjective and irrational concept that has no place in the rational, evidence-based world of medicine. However, this view misunderstands the nature of love and its role in healthcare. Love is not an irrational force that opposes reason; rather, it complements and enhances it. A healthcare provider who practices with love does not abandon scientific principles but instead uses them in the service of human flourishing. Love provides the ethical foundation upon which evidence-based medicine can be practiced with integrity and compassion. It ensures that the pursuit of clinical excellence does not come at the expense of the patient's humanity.

Furthermore, love, in its multiple sociological forms, speaks to the vulnerability of both the patient and the healthcare provider. In the healthcare relationship, both parties are vulnerable—the patient because they are in need of care, and the provider because they must open themselves up to the suffering of another. Love allows for the mutual recognition of this vulnerability and creates a space where healing can occur. It fosters trust, respect, and collaboration, which are essential for effective healthcare delivery. It calls us to view others with “unconditional positive regard” and in order to live this value, it requires us to develop more meaningful relationships with those who we care for.^5^ Recalling Freud's assertion that “Psychoanalysis is, in fact, a cure through love,"^6^ centring love demands that practitioners cultivate humanistic approaches in their practice, thereby expanding their ability to alleviate the suffering associated with illness.

The introduction of love also requires us to acknowledge the specific clinical and professional risks of its misapplication, particularly the danger of a physician's personal ego interfering with patient care. One must also consider another psychoanalytic perspective, that of Jacques Lacan, who viewed love not as a benevolent, unifying force but as an inherently narcissistic illusion. For Lacan, love is fundamentally a misrecognition—a projection of an idealised image onto the patient, driven by the physician's own desire to feel ‘complete’ or fulfilled.^7^ We don't love the other person for who they truly are, but for the fantasised image we have created of them—an image that we believe will complete us and fill a profound lack within our own fragmented selves. The doctor's “love” for the patient is an attempt to project an idealised image onto the patient to feel “complete” or fulfilled themselves. This is why love can be so precarious in a professional context; it risks blurring boundaries and creating a symbiotic, rather than therapeutic, relationship. The doctor who “loves” their patient may, in fact, be seeking to fulfil their own desires for omnipotence or recognition, viewing the patient's recovery not as a goal in itself but as a validation of their own skill. This Lacanian critique suggests that a core concept of love in medicine, if not carefully defined and accompanied by training on professional boundaries, could lead to a dangerous over-identification that prioritises the physician's ego over the patient's well-being, potentially undermining the objectivity essential for effective and ethical care.

## Ethical Implications

Incorporating love into the mission of a medical school would not be without its challenges. The term itself may provoke discomfort or skepticism, as it is often associated with personal relationships rather than professional ones. Furthermore, its subjective nature risks idealisation and misrecognition, potentially leading to destructive outcomes if boundaries are not clearly defined. Unchecked “love” in healthcare risks blurring professional boundaries, fostering favoritism, and undermining objectivity, potentially leading to conflicts of interest or emotional exhaustion. Maintaining strict professional distance and an absolute ban on romantic relationships is crucial to prevent exploitation of power imbalances. History also offers grim examples of “totalitarian love,” where potent emotions are tragically co-opted to serve authoritarian power. This perversion of love demands an unconditional and sublime passion for a leader, where subjects are expected to prioritise this love above all else, including their own lives and the lives of others.^8^ Totalitarian love is an extreme expression of one limitations of love as guiding value in a vocational context and ought to remind us recognise the judicious constraint of personal commitment to negate against burnout.

Love as a value cannot be easily operationalised, developed into a measurable metric or assessed in a standardised format. However, humanistic assessment of student performance, requiring the measurement of complex attitudes and behaviours, is possible and inclusion of medical humanities practice in curricula is already pervasive.^9^ Evidence is accruing of the clinical impacts of empathetic care on clinical outcomes with plausible neurobiological mechanisms elucidated.^10–13^

Operationalising love, while appealing to a metrics-driven healthcare model, would strip it of its essence. To reduce love to measurable behaviours is to risk a perverse reappropriation of the word itself. What we would be measuring is not genuine love, but a hollow, bureaucratic mimicry. This reduction would negate the very relational qualities that make love vital to the healthcare mission, turning a profound human value into just another checkbox.

The transformative power of love as an underpinning value in a curriculum is that it becomes the fundamental lens through which action in practice is viewed. It becomes the culture in which we learn and practice. Within a curriculum of planned educational activities, unless we are clear about our core values, we may fail to make explicit the underlying beliefs and assumptions which motivate our actions.^14^ The Hidden Curriculum often transmits values of cynicism, detachment, and reductionism,^15,16^ which the explicit teaching of love directly counteracts. Love, when properly understood, is not a sentimental or romantic notion but a profound commitment to the well-being of others.

Practicing with love may be thought to threaten our objectivity. Rather than ostracising emotions and negating their influence, it seeks to centre our human responses to suffering.

It calls us to be more than merely virtuous or kind^17^ which can be defined as polite, considerate, or benevolent actions that do not necessarily require a profound emotional or ethical commitment— but to have the humility to recognise our vulnerability as we care for others and witness their suffering. As bell hooks noted: “Taught to believe that the mind, not the heart, is the seat of learning many of us believe that to speak of love with any emotional intensity means that we are perceived as weak and irrational.^18^”

To be effective, love in medicine must be authentic. This authenticity isn't about genuinely “feeling” love, but about using love to fuel an engaged curiosity^19^ in the patient's unique experience. This approach moves beyond the performative aspects of compassion and empathy, grounding the clinician's engagement in a disciplined, humanistic, and ethical exploration of the patient's reality. By doing so, love serves as a guide for professional action, rather than a source of burnout or narcissistic projection.

Some might argue that we should use the term *agape*,^20^ derived from Greek philosophy and a more specific sub-type of love that calls us to deliberately choose to put other's needs ahead of our own, does not require reciprocity, and is semantically distinct from other forms of love such as philia (brotherly love) and eros (romantic love).^21^ However, using a term without a direct translation in modern English could over-complicate our inherent understanding of love. Agape is also often used in Christian theology (for example, Matthew 5:43-46 (Revised Standard Version Bible)) and could be considered too narrow a lens to represent the humanistic values needed in a diverse, secular and inclusive healthcare environment.

## Practical Implications

To transition from advocating for love as a core value to embedding it within medical education, institutions must adopt a multi-faceted approach that addresses mission, curriculum, and assessment. Medical schools should begin by explicitly revising their mission statements to name love, or a close equivalent such as a profound ethical commitment to human dignity, as a primary goal alongside clinical competence, ensuring the value is public and non-negotiable. Concurrently, institutional codes of conduct should be updated to clarify that this professional commitment necessitates the strict maintenance of boundaries, professional distance, and a clear understanding of power dynamics, directly mitigating the risks of misrecognition.

Curricular integration, which specifically targets the affective domain of learning and the development of relational competencies, can be achieved by expanding the use of medical humanities, arts-based learning, and reflective curricula (such as literature, film, art, and narrative medicine exercises).^22^ These interventions do not aim for a formal ‘measurement’ of love but are the essential means by which this value can be embodied in the student and the culture. They offer a safe, protected space to explore human suffering, vulnerability, and ethical complexity. For instance, structured reflective practice,^23^ including Balint groups,^24^ is essential for ‘Self-as-Instrument’ training, cultivating the physician's self-awareness to distinguish authentic commitment from personal ego-driven desire for validation.^25^ By fostering this consistent, reflective engagement, ‘love’ transforms from a theoretical virtue into a disciplined, ethical guide for professional action. Clinical communication training should also be redefined to move beyond procedural empathy, focusing instead on fostering an ‘engaged curiosity’ in the patient's unique experience.^26^

Finally, while operationalising love into a measurable metric is counterproductive, humanistic assessment of complex attitudes and behaviours is possible through direct observation: to evaluate a student's capacity for mutual regard, respect for vulnerability, and ability to establish trust.^9,27^ This should be supported by mandatory student portfolios and peer-review processes that encourage honest reflection on ethical dilemmas and emotional responses to patient care.^23^

Centering love in a medical school's mission would enhance its culture and redefine what it means to be a healthcare professional, creating an environment where compassion, empathy, and respect are not just optional qualities but essential components of professional excellence. This action would influence the professional identity formation of emerging doctors,^28^ providing clear guidance to develop an identity underpinned by ethical commitment to patient care, self awareness and compassionate engagement, while actively counter-acting prevailing models of detached or technocratic medical practice.^29^ Love, as an enabling concept, has the power to transform healthcare by ensuring that the alleviation of suffering is not just a clinical goal but a moral and ethical imperative. As Donabedian asserted, “If you have love, you can then work backward to monitor and improve the system,” underscoring love as fundamental to both quality healthcare and a more humane, just medical system.

**Table:** 

Theory/Concept	Key Figure/Source (references)	Contribution to the Paper's Argument
**"The Secret of Quality is Love"**	Avedis Donabedian^1^	Serves as the paper's foundation, advocating for an ethical commitment (love) that complements healthcare systems design and process monitoring and provides a counterbalance to reductionistic models of medicine
**Client-Centered Therapy**	Carl Rogers ^5^	Introduces the concept of “unconditional positive regard,” which supports the argument that love fosters trust, respect, and mutual regard in the patient-provider relationship.
**Psychoanalysis (Transference- Love)**	Sigmund Freud ^6^	Quoted assertion: “Psychoanalysis is, in fact, a cure through love,” which is used to support the idea that centering love demands humanistic approaches to alleviate suffering.
**Psychoanalysis (Love as Narcissistic Illusion)**	Jacques Lacan ^7^	Provides the central critique of love in medicine, arguing it is a misrecognition or projection that risks prioritising the physician's ego/desires over the patient's well-being and blurring professional boundaries.
**Totalitarian Love**	Slavoj Žižek ^8^	Cited as a grim, extreme example of the perversion and limitations of love as a guiding value in a vocational context, underscoring the need for judicious constraint of personal commitment.
**Critique of Emotional Weakness**	bell hooks ^18^	Quoted to highlight the societal bias that views speaking of love or emotional intensity as “weak and irrational,” which the paper seeks to challenge by centering human responses to suffering.
**Virtues versus love**	Aristotle, Nicomachean Ethics^30^	*Nicomachean Ethics* defines virtue (and therefore virtues like kindness) not as a mere feeling or polite action, but as a disposition or state of character. The distinction between “polite, considerate actions” (kindness) and a “profound emotional or ethical commitment” (love) is a classic theme in philosophical ethics, where a spontaneous action is contrasted with an act driven by a fully rationalised, stable, and committed disposition.
***Agape* (Greek & Christian Concept of Love)**	C. S. Lewis ^21^, Greek Lexicon	Discussed as a specific type of love (unconditional, non-reciprocal) that is ultimately rejected by the paper for being linguistically complex and too narrow for a diverse, secular healthcare environment.

## References

[1] Ayanian JZ Markel H . Donabedian’s lasting framework for health care quality. N Engl J Med. 2016;375(3):205-207. 27468057 10.1056/NEJMp1605101

[2] Lown BA Rosen J Marttila J . An agenda for improving compassionate care: a survey shows about half of patients say such care is missing. Health Aff. 2011;30(9):1772-1778. 10.1377/hlthaff.2011.0539 21900669

[3] Ahmed Z Ellahham S Soomro M Shams S Latif K . Exploring the impact of compassion and leadership on patient safety and quality in healthcare systems: a narrative review. BMJ open quality. 2024;13(Suppl 2):e002651. 10.1136/bmjoq-2023-002651 PMC11086414 38719520

[4] Miles A . On a medicine of the whole person: away from scientistic reductionism and towards the embrace of the complex in clinical practice. J Eval Clin Pract. 2009;15(6). 10.1111/j.1365-2753.2009.01354.x 20367688

[5] Rogers CR . Client-centered therapy: Its current practice, implications, and theory. Houghton Mifflin Boston; 1951.

[6] Freud S . Observations on transference-love. SE. 1915;12:157-171. PMC3330334 22700141

[7] Jacques L Alan S . Écrits: A Selection. WW Norton; 1977.

[8] Zizek S Žižek S . Did somebody say totalitarianism?: five interventions in the (mis) use of a notion. Verso; 2002.

[9] Buck E Holden M Szauter K . A methodological review of the assessment of humanism in medical students. Acad Med. 2015;90(11):S14-S23. 10.1097/ACM.0000000000000910 26505097

[10] Hojat M . Empathy in health professions education and patient care. 2016.

[11] Hojat M Maio V Pohl CA Gonnella JS . Clinical empathy: definition, measurement, correlates, group differences, erosion, enhancement, and healthcare outcomes. Discover Health Systems. 2023;2(1):8.

[12] Villa G Lanini I Amass T , et al. Effects of psychological interventions on anxiety and pain in patients undergoing major elective abdominal surgery: a systematic review. Perioperative Medicine. 2020;9(1):38. 33292558 10.1186/s13741-020-00169-x PMC7722323

[13] Malenfant S Jaggi P Hayden KA Sinclair S . Compassion in healthcare: an updated scoping review of the literature. BMC Palliat Care. 2022;21(1):80. 35585622 10.1186/s12904-022-00942-3 PMC9116004

[14] Hafferty FW Franks R . The hidden curriculum, ethics teaching, and the structure of medical education. Acad Med. 1994;69(11):861-871. 7945681 10.1097/00001888-199411000-00001

[15] Hershey MS Stoddard HA . A scoping review of research into the origins of cynicism among medical trainees. Med Sci Educ. 2021;31(4):1511-1517. 34457989 10.1007/s40670-021-01328-5 PMC8368361

[16] Kopelman L . Cynicism among medical students. Jama. 1983;250(15):2006-2010. 6620501

[17] Greco A González-Ortiz LG Gabutti L Lumera D . What's the role of kindness in the healthcare context? A scoping review. BMC Health Serv Res. 2025;25(1):207. 39910597 10.1186/s12913-025-12328-1 PMC11796266

[18] Hooks B . All about love: New visions. 2000.

[19] Guidi C Traversa C . Empathy in patient care: from ‘clinical empathy’to ‘empathic concern’. Medicine, Health Care and Philosophy. 2021;24(4):573-585. 34196934 10.1007/s11019-021-10033-4 PMC8557158

[20] Liddell HG . A greek-english lexicon: at the Clarendon Press; 1925.

[21] Lewis C . The four loves: An exploration of the nature of love. Mariner Books; 1960.

[22] Powley E Higson R . The Arts in Medical Education: a practical guide. CRC Press; 2017.

[23] Uygur J Stuart E De Paor M , et al. A best evidence in medical education systematic review to determine the most effective teaching methods that develop reflection in medical students: BEME guide No. 51. Med Teach. 2019;41(1):3-16. 30634872 10.1080/0142159X.2018.1505037

[24] Monk A Hind D Crimlisk H . Balint groups in undergraduate medical education: a systematic review. Psychoanal Psychother. 2018;32(1):61-86.

[25] Mann K Gordon J MacLeod A . Reflection and reflective practice in health professions education: a systematic review. Advances in Health Sciences Education. 2009;14(4):595-621. 18034364 10.1007/s10459-007-9090-2

[26] Dyche L Epstein RM . Curiosity and medical education. Med Educ. 2011;45(7):663-668. 21649698 10.1111/j.1365-2923.2011.03944.x

[27] Misch DA . Evaluating Physicians’ professionalism and humanism: the case for humanism “connoisseurs”. Acad Med. 2002;77(6):489-495. 12063192 10.1097/00001888-200206000-00004

[28] Cruess RL Cruess SR Boudreau JD Snell L Steinert Y . Reframing medical education to support professional identity formation. Acad Med. 2014;89(11):1446-1451. 25054423 10.1097/ACM.0000000000000427

[29] Kim DT Applewhite MK Shelton W . Professional identity formation in medical education: some virtue-based insights. Teach Learn Med. 2024;36(3):399-409. 37140086 10.1080/10401334.2023.2209067

[30] Pakaluk M . Nicomachean ethics: Books VIII and IX. OUP Oxford; 1998.

